# Gender inequities in patterns of authorship contributions in dentistry: an observational study

**DOI:** 10.21142/2523-2754-1402-2026-288

**Published:** 2026-04-04

**Authors:** Laura Barreto Moreno, Cristina Helena Morello Sartori, Sarah Arangurem Karam, Marcos Britto Correa, Françoise Hélène van de Sande, Anelise Fernandes Montagner

**Affiliations:** 1 Graduate Program of Dentistry, Federal University of Pelotas. Pelotas, Brazil. laurab4moreno@gmail.com marcosbrittocorrea@hotmail.com fvandesande@gmail.com animontag@gmail.com Universidade Federal de Pelotas Graduate Program of Dentistry Federal University of Pelotas Pelotas Brazil laurab4moreno@gmail.com marcosbrittocorrea@hotmail.com fvandesande@gmail.com animontag@gmail.com; 2 School of Dentistry, Federal University of Pelotas. Pelotas, Brazil. crissartori0028@gmail.com Universidade Federal de Pelotas School of Dentistry Federal University of Pelotas Pelotas Brazil crissartori0028@gmail.com; 3 Professional Master's Degree in Health in the Life Cycle, Catholic University of Pelotas. Pelotas, Brazil. arangurem72@gmail.com Universidade Federal de Pelotas Professional Master's Degree in Health in the Life Cycle Catholic University of Pelotas Pelotas Brazil arangurem72@gmail.com

**Keywords:** gender, dentistry, gender inequities, authorship, data collection, género, odontología, inequidades de género, autoría, recopilación de datos

## Abstract

**Objective:**

This observational study investigated gender differences in authorship contributions in high-impact, multidisciplinary dentistry journals.

**Material and Methods:**

A sample of 396 articles published between January 1 and December 31, 2023, across the five high-impact multidisciplinary journals in the dentistry field was analyzed. The selection of journals considered the impact factor in the Journal Citation Reports 2023 in the dentistry field. It includes the five dental multidisciplinary journals with the highest impact factor, considering journals with multidisciplinary dentistry subjects. Journals with a specific focus on dental areas or multidisciplinary journals covering the same area were excluded. The binary gender of the first and last authors was identified using the Genderize database, and contribution roles were extracted from the authors' statements. Data analysis included descriptive statistics and Pearson’s chi-squared tests.

**Results:**

Men were the majority in both first (57.1%) and last (65.7%) authorship positions. Among first authors, significant gender differences were found: women were more involved in data collection (53.9% vs. 46.1%, p = 0.001) and more likely to contribute to all five authorship categories (p = 0.04). No significant gender differences were observed among the last authors. Geographical variation was noted, with South America showing a higher proportion of women as first authors (64.4%).

**Conclusion:**

Overall, the study reveals gender disparities in the distribution of scientific labor, particularly in the position of first authorship, and highlights the need for greater equity and transparency in recognizing research contributions.

## RESUMEN

**Objetivo:**

Este estudio observacional investigó las diferencias de género en las contribuciones de autoría en revistas multidisciplinarias de odontología de alto impacto.

**Materiales y métodos:**

Se analizó una muestra de 396 artículos publicados entre el 1 de enero y el 31 de diciembre de 2023 en cinco revistas multidisciplinarias de alto impacto en el campo de la odontología. La selección de revistas se basó en el factor de impacto del Journal Citation Reports 2023 en el campo de la odontología. Se incluyeron las cinco revistas multidisciplinarias de odontología con el mayor factor de impacto, considerando aquellas que abarcan temas multidisciplinarios de odontología. Se excluyeron las revistas con un enfoque específico en áreas odontológicas o las revistas multidisciplinarias que cubren la misma área. El género binario del primer y último autor se identificó mediante la base de datos Genderize, y los roles de contribución se extrajeron de las declaraciones de los autores. El análisis de datos incluyó estadística descriptiva y pruebas de chi-cuadrado de Pearson.

**Resultados:**

Los hombres fueron mayoría tanto en la primera (57,1%) como en la última (65,7%) autoría. Entre los primeros autores, se encontraron diferencias significativas por género: las mujeres participaron más en la recopilación de datos (53,9% frente al 46,1%, p = 0,001) y tuvieron mayor probabilidad de contribuir en las cinco categorías de autoría (p = 0,04). No se observaron diferencias significativas por género entre los últimos autores. Se observó variación geográfica, con una mayor proporción de mujeres como primeras autoras en Sudamérica (64,4%).

**Conclusión:**

En general, el estudio revela disparidades de género en la distribución del trabajo científico, particularmente en la autoría principal, y destaca la necesidad de mayor equidad y transparencia en el reconocimiento de las contribuciones a la investigación.

## INTRODUCTION

Gender disparities have been deeply embedded in societal structures throughout various sectors ^1^, and the global scientific community is no exception. Despite noticeable progress in women's representation across many academic disciplines, the dominance of men in research and academia remains pervasive ^2^. This pattern also persists in dentistry, with recent studies demonstrating a significant underrepresentation of women among the authors of the most-cited articles in dentistry ^3^^,^^4^. Furthermore, this trend has not shown substantial changes over the last four decades in terms of gender distribution in published articles in more prestigious journals ^5^ and receiving a higher number of citations than their female counterparts, not only in dentistry but in several fields ^1^^,^^6^. Moreover, these inequities are particularly evident in leading scientific dental journals, where women remain underrepresented in authorship, review, and editorial roles ^7^^-^^12^.

Research highlights persistent gender disparities in academic success and recognition, underscoring the additional barriers women face in advancing their careers compared to men ^13^. The underrepresentation of women in leadership roles within dentistry ^3^^-^^5^ and in senior academic positions reflects a complex issue shaped by structural, institutional, and cultural factors ^14^.

The distribution of work and recognition among authors in academic publishing has garnered significant attention in recent years. Examining authorship through the lens of gender reveals nuanced patterns of representation within the academic sphere ^15^. Authorship in scientific publishing serves as a critical measure of academic recognition, directly influencing researchers' career progression, funding opportunities, and professional visibility. As research has become increasingly collaborative, analyzing authorship patterns through a gendered perspective is essential to understanding the distribution of contributions and recognition in academia ^10^^,^^16^.

An analysis noted that female authors are more likely to be placed second, even when both authors report they have contributed equally ^17^. The authorship order in scientific articles reflects not only individual involvement but also norms and power dynamics within academic collaborations ^18^^,^^19^. Proper attributions of authorship are crucial for ensuring that researchers receive appropriate credit for their work, impacting their academic trajectories and future opportunities ^20^^,^^21^.

Since authorship conveys academic, social, and financial benefits while implying responsibility for published work, the International Committee of Medical Journal Editors (ICMJE) advocates for transparent statements of author contributions ^22^. Many journals now require these statements to clarify individual contributions, promoting transparency in scholarly publishing and recognizing each individual's contributions to the study ^23^.

Considering the persistent gender inequities in academia, it is plausible that such differences might also extend to the authorship patterns and the contribution roles of each author in scientific publishing. Therefore, this observational study aims to evaluate the association between authors' gender and their patterns of contributions to authorship in articles published in high-impact, multidisciplinary dentistry journals. 

## MATERIALS AND METHODS

### Study design

The full protocol and data are available online at the Open Science Framework platform (link: https://osf.io/npu3x/). This cross-sectional observational study was reported according to the Strengthening the Reporting of Observational Studies in Epidemiology Statement (STROBE) ^24^ (Table 1). No ethical approval was required to develop this study, as all information is publicly available. 

**Table 1:** STROBE Statement - Checklist of items that should be included in reports of cross-sectional studies

	Item	Recommendation	Page
Title and abstract	1	(a) Indicate the study’s design with a commonly used term in the title or the abstract	1
		(b) Provide in the abstract an informative and balanced summary of what was done and what was found	1
Introduction			
Background/rationale	2	Explain the scientific background and rationale for the investigation being reported	2
Objectives	3	State specific objectives, including any prespecified hypotheses	2
Methods			
Study design	4	Present key elements of study design early in the paper	3
Setting	5	Describe the setting, locations, and relevant dates, including periods of recruitment, exposure, follow-up, and data collection	4
Participants	6	(a) Give the eligibility criteria, and the sources and methods of selection of participants	4
Variables	7	Clearly define all outcomes, exposures, predictors, potential confounders, and effect modifiers. Give diagnostic criteria, if applicable	4-5
Data sources/ measurement	8*	For each variable of interest, give sources of data and details of methods of assessment (measurement). Describe comparability of assessment methods if there is more than one group	4
Bias	9	Describe any efforts to address potential sources of bias	4
Study size	10	Explain how the study size was arrived at	
Quantitative variables	11	Explain how quantitative variables were handled in the analyses. If applicable, describe which groupings were chosen and why	5
Statistical methods	12	(a) Describe all statistical methods, including those used to control for confounding	5
		(b) Describe any methods used to examine subgroups and interactions	5
		(c) Explain how missing data were addressed	5
		(d) If applicable, describe analytical methods taking account of sampling strategy	-
		(e) Describe any sensitivity analyses	-
Results			
Participants	13*	(a) Report numbers of individuals at each stage of study-eg numbers potentially eligible, examined for eligibility, confirmed eligible, included in the study, completing follow-up, and analysed	5
		(b) Give reasons for non-participation at each stage	5
		(c) Consider use of a flow diagram	-
Descriptive data	14*	(a) Give characteristics of study participants (eg demographic, clinical, social) and information on exposures and potential confounders	5
		(b) Indicate number of participants with missing data for each variable of interest	5
Outcome data	15*	Report numbers of outcome events or summary measures	5-6-7
Main results	16	(a) Give unadjusted estimates and, if applicable, confounder-adjusted estimates and their precision (eg, 95% confidence interval). Make clear which confounders were adjusted for and why they were included	5-6-7
		(b) Report category boundaries when continuous variables were categorized	-
		(c) If relevant, consider translating estimates of relative risk into absolute risk for a meaningful time period	-
Other analyses	17	Report other analyses done-eg analyses of subgroups and interactions, and sensitivity analyses	-
Discussion			
Key results	18	Summarise key results with reference to study objectives	7
Limitations	19	Discuss limitations of the study, taking into account sources of potential bias or imprecision. Discuss both direction and magnitude of any potential bias	9
Interpretation	20	Give a cautious overall interpretation of results considering objectives, limitations, multiplicity of analyses, results from similar studies, and other relevant evidence	8-9
Generalizability	21	Discuss the generalisability (external validity) of the study results	8-9
Other information			
Funding	22	Give the source of funding and the role of the funders for the present study and, if applicable, for the original study on which the present article is based	10

Deviations from protocol: It was not possible to include data from 2013 and 2018 as originally planned, as most articles from those years did not provide authorship contribution details.

### Journal eligibility criteria

The selection of journals considered the impact factor in the Journal Citation Reports 2023 in the dentistry field. It includes the five dental multidisciplinary journals with the highest impact factor, considering journals with multidisciplinary dentistry subjects. Journals with a specific focus on dental areas or multidisciplinary journals covering the same area were excluded (Table 2). The following journals were included: International Journal of Oral Science (IF: 14.9), Journal of Dental Research (IF: 8.9), Journal of Dentistry (IF: 4.4), Journal of the American Dental Association (IF: 3.6), and Clinical Oral Investigations (IF: 3.6). 

**Table 2:** Top-ranked journals in the Dentistry, Oral Surgery & Medicine field in the Journal Citation Reports and their reasons for being excluded in the present study

Journal	Impact Factor	Reason for exclusion
Periodontology 2000	18.6	Publish only literature reviews.
Journal of Clinical Periodontology	6.7	Not a multidisciplinary journal
Japanese Dental Science Review	6.6	Publish only literature reviews.
International Endodontic Journal	5.0	Not a multidisciplinary journal
Dental Materials	5.0	Not a multidisciplinary journal
Progress in Orthodontics	4.8	Not a multidisciplinary journal
Oral Oncology	4.8	Not a multidisciplinary journal
Journal of Prosthetic Dentistry	4.6	Not a multidisciplinary journal
Journal of Periodontology	4.3	Not a multidisciplinary journal
Clinical Oral Implant Research	4.3	Not a multidisciplinary journal
Journal of Endodontics	4.3	Not a multidisciplinary journal
Seminars in Orthodontics	4.2	Not a multidisciplinary journal
Caries Research	4.2	Not a multidisciplinary journal
Journal of Prosthodontics - Implant Esthetics and Reconstructive Dentistry	4.0	Not a multidisciplinary journal
Oral Diseases	3.8	Not a multidisciplinary journal
International Journal of Paediatric Dentistry	3.8	Not a multidisciplinary journal
Molecular Oral Microbiology	3.7	Not a multidisciplinary journal
Journal of Prosthodontics Research	3.6	Not a multidisciplinary journal
Clinical and Implant Dentistry and Related Research	3.6	Not a multidisciplinary journal
European Journal of Paediatric Dentistry	3.6	Not a multidisciplinary journal
Journal of Periodontal Research	3.5	Not a multidisciplinary journal
Journal of Dental Sciences	3.5	Publish only literature reviews.

### Sample size

A sample size calculation was performed to determine the number of articles to be included. Considering a mean of 1.250 articles published annually in the five journals, a hypothetical gender frequency of 50%, a 5% error margin, and a 95% confidence interval (CI), at least 300 articles would be necessary.

### Search and selection

The search strategy did not consider any combination of MeSH (Medical Subject Headings) terms and free-text words. The search was conducted in January 2024 at the SCOPUS database. The search strategy considered limits: inserting the ISBN of each journal (ISSN IJOS = 2049-3169, ISSN JDR = 1544-0591, ISSN JoD = 0300-5712, ISSN JADA = 0002-8177, and ISSN CLOI = 1436-3771) and the year of interest (2023). A list comprising all articles published in the selected journals in 2023 was generated, from which an aleatory sample was selected for inclusion.

Two reviewers (LBM and CRMS) performed the study selection and screened independently all full texts to determine if they satisfied the inclusion criteria. Discrepancies in screening full texts were solved through a discussion between the two reviewers with the help of a third reviewer (AFM), if necessary. 

### Articles eligibility criteria

Dental articles published between January 1 and December 31, 2023, in the five selected journals, with any study design, that reported an authorship contribution statement were included. It considered only the first submission of a manuscript. It excluded errata, commentaries, notes, editorials, perspectives, brief and short communications, and other types of papers not considered typical full‐length research studies. Moreover, manuscripts published by only one author were also excluded.

### Data extraction

Two reviewers (LBM and CRMS) extracted the data from each included study into predesigned coding sheets: [1] Study identification: first and last author’s full names, first and last author’s full genders (woman and man), location/country of the corresponding author, year of publication, journal name, and journal impact factor. [2] Author contribution: reported information was collected from the manuscript, considering the International Committee of Journal Medical Editors (ICJME) criteria for defining the role of authors and contributors: a. Conception / idea /design of the work; b. Data collection; c. Data analysis / interpretation; d. Wrote the manuscript; e. Critically review the manuscript; f. All five contributions. [3] Funding information (Yes/No/Not reported), and type of funding (public/private); [4] Open access (yes or subscription).

### Assignment of authors’ gender 

The binary gender of the authors was determined by associating their first names with the probability of the name being held by a man versus by a woman, using the online Genderize database (https://api.genderize.io/?name=). If an author’s name was not listed in genderize.io or was listed but had less than a 90% probability of being one gender, it was used in an Internet search to determine gender, checking applicants’ online CVs (e.g. ORCID, University websites, Research Gate). 

### Data synthesis

Data were summarized in descriptive tables to present the distribution of variables (continent, type of article access, funding report, funding type, and author contributions) according to the gender of the first and last authors, using Pearson's chi-squared test. The frequencies of the first and last authors' genders, based on their contributions, were presented using equiplots (http://www.equidade.org/equiplot). The analyses were performed with the Stata version 15.0 statistical software program, considering a significance level of 5%.

## RESULTS

Table 3 presents the gender distribution of the first and last authors and their contributions to the analyzed articles. Of the full list of 1.059 eligible studies, 396 were aleatorially selected. Among the included studies, men were the majority in both authorship positions, accounting for 57.1% and 65.7% of first and last authorship positions, respectively. Of the 792 identified authors, 61.4% (n = 485) were men occupying the first and last authorship positions in the articles (data not shown in tables).

**Table 3:** Frequencies of gender distribution and authorship contributions among the first and last authors

	First Authorship N (%)	Last Authorship N (%)
Author's gender		
Woman	170 (42.9)	135 (34.3)
Man	226 (57.1)	259 (65.7)
Contribution		
Conception/ idea /design of the work	265 (66.8)	164 (41.3)
Data collection	141 (35.5)	53 (13.4)
Analysis and interpretation	298 (75.1)	180 (45.3)
Wrote the manuscript	276 (69.5)	142 (35.8)
Critically review the manuscript	218 (54.9)	353 (88.9)
All five contributions	18 (4.5)	11 (2.8)

It was observed that the last authors primarily performed supervisory roles, with critical manuscript review being the most frequent contribution (88.9%). In contrast, their participation in operational activities, such as data collection and analysis, was less frequent.

First authors, on the other hand, demonstrated a greater diversity of contributions, with the most prevalent contribution concentrated in data analysis and interpretation (75.1%), manuscript first drafting (69.5%), study conception and design (66.8%), and data collection (35.5%). First authors were more involved in all five activities (4.5%) compared to last authors (2.8%). 

Table 4 presents the descriptive analysis of first authorship by gender, considering the covariates. A significant difference was observed among continents (p < 0.001), with women representing the minority in all continents, except in South America, where they are the majority (64.4%; 95% CI: 52.6-74.6). A significant difference was also found concerning funding report (p = 0.02): although men still comprised the majority of first authors in funded studies (54.0%; 95% CI: 48.4-59.5), the proportion of female first authors was notably higher in studies that reported funding (46.0%) compared to those without funding (23.3%), effectively doubling. No significant differences were observed for funding type (p = 0.49) or open access (p = 0.68).

**Table 4:** Descriptive analysis of the first author's gender

	First Author Gender			
	Woman % (95% CI)	Man % (95% CI)	p-value
Continent			<0.001
Asia	36.0 (28.4-44.3)	64.0 (55.6-71.6)	
Europe	44.7 (36.1-53.7)	55.3 (46.3-63.9)	
North America	29.3 (18.9-42.5)	70.7 (57.5-81.1)	
South America	64.4 (52.6-74.6)	35.6 (25.4-47.4)	
Oceania	33.3 (0.3-98.9)	66.7 (1.0-99.7)	
Open access			0.68
No	41.8 (34.5-49.4)	58.2 (50.6-65.5)	
Yes	43.8 (37.4-50.4)	56.2 (49.6-62.6)	
Funding Report			0.02
No report	40.5 (26.5-56.2)	59.5 (43.8-73.5)	
No	23.3 (12.7-38.5)	76.7 (61.4-87.2)	
Yes	46.0 (40.5-51.6)	54.0 (48.4-59.5)	
Funding Type			0.49
Private	42.2 (33.9-50.9)	57.8 (49.0-66.1)	
Public	47.6 (40.4-54.8)	52.4 (45.2-59.6)	
Private and Public	66.7 (1.0-99.7)	33.3 (0.3-98.9)	
Author's contribution			
Conception/ idea /design of the work			0.94
No	43.2 (34.9-51.8)	56.8 (48.2-65.1)	
Yes	42.8 (36.9-48.9)	57.2 (51.1-63.1)	
Data collection			0.001
No	36.9 (31.1-42.9)	63.1 (57.0-68.9)	
Yes	53.9 (45.6-62.0)	46.1 (37.9-54.4)	
Analysis and interpretation			0.49
No	45.9 (36.2-55.9)	54.1 (44.1-63.8)	
Yes	41.9 (36.4-47.6)	58.1 (52.3-63.6)	
Wrote the manuscript			0.44
No	40.0 (31.5-49.1)	60.0 (50.9-68.4)	
Yes	44.2 (38.4-50.1)	55.8 (49.8-61.6)	
Critically review the manuscript			0.27
No	39.9 (32.9-47.3)	60.1 (52.7-67.1)	
Yes	45.4 (38.9-52.1)	54.6 (47.9-61.1)	
All five contributions			0.04
No	41.8 (36.9-46.8)	58.2 (53.1-63.1)	
Yes	66.7 (41.0-85.2)	33.3 (14.8-58.9)	

Footnotes: 95% CI: 95% Confidence Interval

Regarding authorship contributions, a significant association was observed between first author gender and participation in data collection (p = 0.001), with women more frequently reporting involvement (53.9%; 95% CI: 45.6-62.0) than men (46.1%; 95% CI: 37.9-54.4). Likewise, women were more likely to be involved in all five contributions (p = 0.04). No significant gender differences were observed for the other types of contributions for the first authorship (all p > 0.05) (Table 4).

Table 5 presents the results of the descriptive analysis of the last authorship by gender, considering the covariates. For the last authorship position, no variables showed a statistically significant association with gender (all p > 0.05). Although the gender distribution did not differ significantly among the continents (p = 0.34), South America had the highest proportion of women as the last author (42.5%, 95% CI: 31.5-54.2%), compared to other regions such as North America and Asia, where men were predominant. No significant differences were observed regarding open access (p = 0.44), funding report (p = 0.09), or funding type (p = 0.47).

**Table 5:** Descriptive analysis of the last author's gender

	Last Author Gender		
	Woman % (95% CI)	Man % (95% CI)	p-value
Continent			0.34
Asia	32.6 (25.3-40.9)	67.4 (59.1-74.7)	
Europe	34.4 (26.3-43.4)	65.6 (56.6-73.5)	
North America	29.3 (18.9-42.5)	70.7 (57.5-81.1)	
South America	42.5 (31.5-54.2)	57.5 (45.8-68.5)	
Oceania	0.0 (-)	100.0 (-)	
Open access			0.44
No	32.1 (25.5-39.6)	67.9 (60.4-74.5)	
Yes	35.8 (29.8-42.3)	64.2 (57.6-70.2)	
Funding Report			0.09
No report	24.4 (13.4-40.2)	75.6 (59.8-86.6)	
No	23.8 (13.1-39.4)	76.2 (60.6-86.9)	
Yes	37.0 (31.8-42.5)	63.0 (57.5-68.2)	
Funding Type			0.47
Private	34.6 (26.8-43.4)	65.4 (56.6-73.2)	
Public	37.8 (31.1-45.1)	62.2 (54.9-68.9)	
Private and Public	66.7 (1.0-99.7)	33.3 (0.2-98.9)	
Author's contribution			
Conception/ idea /design of the work			0.41
No	32.6 (26.8-38.9)	67.4 (61.0-73.2)	
Yes	36.6 (29.5-44.3)	63.4 (55.7-70.5)	
Data collection			0.96
No	34.3 (29.4-39.5)	65.7 (60.5-70.6)	
Yes	34.0 (22.3-47.9)	66.0 (52.1-77.7)	
Analysis and interpretation			0.32
No	32.1 (26.2-38.7)	67.9 (61.3-73.8)	
Yes	36.9 (30.1-44.2)	63.1 (55.8-69.9)	
Wrote the manuscript			0.41
No	32.8 (27.3-38.9)	67.2 (61.1-72.7)	
Yes	36.9 (29.3-45.2)	63.1 (54.8-70.7)	
Critically review the manuscript			0.76
No	36.4 (23.3-51.8)	63.6 (48.2-76.7)	
Yes	34.0 (29.2-39.1)	66.0 (60.8-70.9)	
All five contributions			0.88
No	34.2 (29.6-39.1)	65.8 (60.9-70.4)	
Yes	36.4 (12.4-69.8)	63.6 (30.2-87.6)	

Footnotes: 95% CI: 95% Confidence Interval

Regarding specific contributions, there were no significant gender differences for any of the evaluated authorship contribution roles. Both genders participated similarly in conception/design (p = 0.41), data collection (p = 0.96), analysis and interpretation (p = 0.32), manuscript writing (p = 0.41), and critical review (p = 0.76). The higher absolute percentage of men in each contribution reflects their predominance in the last authorship position overall, as also illustrated in Figure 2.

**Figure 1:**
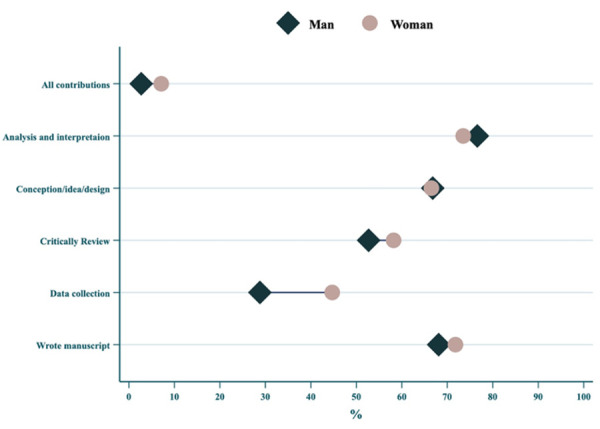
Frequency of contributions reported by first authors, stratified by gender.

Figure 1 illustrates the frequency of contribution reported by first authors, stratified by gender, highlighting marked differences in the patterns of contributions between genders, since women more frequently extracted data compared to men. The most reported contributions by first authors were data analysis and interpretation, study conception and design, and manuscript writing. Women reported participating in data collection more frequently than men. Contributions related to administrative or technical aspects appeared less frequently for both genders.

**Figure 2:**
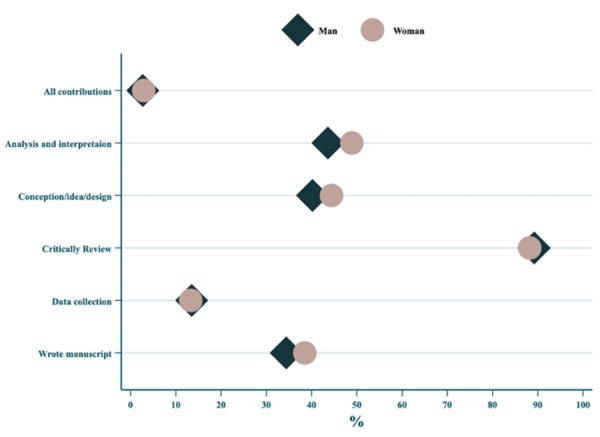
Frequency of contributions reported by last authors, stratified by gender.

Figure 2 illustrates the frequency of contribution patterns made by the last authors across publications, stratified by gender. The figure shows the similarity in the patterns of contributions in both genders. Critical review stands out as the most frequent contribution, while participation in more practical stages, such as data collection and first draft writing, appears less frequently for both genders.

## DISCUSSION

This study examines the intricate dynamics of authorship contributions through the lens of gender, shedding light on representation patterns within scholarly publishing. In particular, the interest was in trends involving the sharing of contributions among authors differing in gender, since inequities in distribution could translate into differences in gender recognition for scientific accomplishment. The findings reveal gender inequities in academic authorship contributions in scientific publications, considering first authorship, and the predominance of men authors in the first and last authorship positions. 

Although the predominance of men in first and last authorship positions remains a consistent pattern in academic publishing ^5^, understanding how research contributions are distributed among authors is important to inform policies that promote equitable recognition in science. Clear documentation of individual contributions, such as through the Contributor Roles Taxonomy (CRediT), has been proposed to improve transparency and fairness in the attribution of scientific work ^23^^,^^25^^,^^26^. In this study, the first authors were most frequently involved in study conception (66.8%), data analysis (75.1%), and manuscript writing (69.5%). A significant gender difference was observed in data collection, with women more frequently reporting involvement in this activity compared to men. Women first authors were also more likely to report having participated in all five contribution categories.

These findings are consistent with previous data indicating that women often contribute to a broader range of research activities, such as contributions related to the technical and planning stages, but are less frequently credited in supervisory or leadership roles ^27^. Overall, this indicates that first authors, regardless of gender, are more engaged in technical and analytical aspects of the research, while tasks associated with administrative or supervisory roles were reported less frequently. This aligns with prior evidence suggesting that early-career researchers-often listed as first authors-are more likely to perform the core research tasks, whereas senior authors are involved in oversight and review ^28^^,^^29^.

In contrast, the contribution pattern among the last authorship differed. The most frequently reported activity was critical manuscript review (88.9%), with limited involvement in practical stages such as data collection and initial drafting. No significant gender differences were observed in contribution patterns among the last authors. These findings align with the traditional role of senior authors in scientific publishing, often associated with project supervision, coordination, and final approval of the manuscript ^28^^,^^29^. The results suggest a clear division of responsibilities between authorship positions, with first authors performing a broader range of research tasks and last authors primarily involved in supervisory roles ^30^.

While contribution patterns varied by authorship position, gender-related differences were identified only among first authors. Prior studies have shown that women are more likely to experience authorship disputes and feel their contributions are undervalued, particularly in collaborative environments ^31^. Women also tend to initiate authorship discussions earlier in the research process, while men may make unilateral decisions at later stages, potentially reinforcing recognition disparities. Moreover, articles led by male authors are less likely to include female co-authors, limiting women's visibility and collaboration opportunities ^3^^,^^10^. Even when women contribute significantly to research, they are less frequently credited across various roles, including supervision and critical review, which may negatively affect team dynamics and reinforce structural barriers to leadership positions ^30^^,^^32^.

The predominance of men in both first (57.1%) and last (65.7%) authorship positions highlights persistent gender inequality in academic publishing, particularly in roles associated with scientific leadership and supervision. These findings reflect the ongoing underrepresentation of women in prominent authorship roles ^10^, and align with previous literature indicating that men are more frequently recognized in prominent authorship positions ^3^^-^^5^. Last authorship, in particular, is widely recognized as a marker of scientific authority and seniority, areas in which women remain disproportionately absent. This pattern aligns with previous studies that have identified structural and social barriers to women’s academic advancement, especially in contexts where authorship order is linked to influence and recognition within the research team ^17^. Although multiple factors contribute to these trends, one explanation is that women may receive less credit for their scientific contributions, particularly in leadership-related roles ^33^.

The findings showed a significant association between first author gender and the presence of funding (p = 0.02). Although men remained the majority of first authors in funded studies (54.0%), the proportion of women was notably higher in studies that reported funding (46.0%) compared to those without funding (23.3%), representing a twofold increase. However, no significant gender differences were observed in last authorship according to funding status. Systematic reviews indicate that funding gives women a greater opportunity to lead research, reflected in their visibility as lead authors ^14^^,^^19^. Access to research funding is associated with participation in leading authorship roles and can influence gender dynamics in academic productivity and visibility. Evidence from transdisciplinary fields such as sustainability also suggests that funding structures that encourage diverse collaborations may foster greater inclusion of women in research teams ^34^. Similar patterns have been observed in biological and medical sciences, where women’s participation in funded projects is often associated with improved representation in authorship ^35^. Nonetheless, the influence of funding on gender equity is shaped by multiple contextual factors, including disciplinary norms and institutional priorities. Greater inclusion of women in academic leadership has been associated with enhanced scientific quality, innovation, and decision-making ^36^. Diverse teams tend to be more effective, bringing broader perspectives and improved organizational outcomes ^38^.

In addition, the differences between continents regarding scientific authorship are striking and reveal significant gender disparities. The higher female representation among first authors in South America (64.4%), as observed by Braz et al. ^28^ can be attributed to local policies that promote gender equity, such as quota systems and advancement initiatives ^19^^,^^38^. Similarly, it is possible to affirm that women account for approximately 39.76% of first authors and 30% of last authors in oncology publications, reflecting a significant female presence in the South American region. In contrast, regions such as Asia continue to show a predominance of men in authorship positions. According to the same study, the proportions of women as first and last authors in Asia were 42.73% and 32.27%, respectively ^39^. These findings reinforce the importance of culturally contextualized approaches to promote greater inclusion and gender equity in scientific authorship, taking into account regional and sociocultural specificities.

The present study has some limitations, as the tool used (Genderize) considers gender as a binary variable (woman or man) and disregards people with other gender identities, such as nonbinary individuals ^40^. This means that gender identity and expression are not limited to males or females, including, for example, transgender people who feel their gender cannot be defined within the margins of the gender binary. Thus, information on gender bias may have been lost by not considering gender-diverse people. Also, the determination of gender turns out not to be completely accurate since several names can belong to both genders. Another limitation is that the authorship of articles is only one of many indicators of research activity and contribution, and that the related contribution is reported by authors, although integrity and ethics are expected in its reporting, it does not guarantee the real extent of contribution by each author. Furthermore, it is important to recognize that power structures in academia are intersectional. Gender is only one dimension of inequity and discrimination; other factors, such as race, sexual orientation, socioeconomic status, nationality, and others, also influence these dynamics. 

Although the sample of this study is selective, it strengthens internal validity by reducing variations across specialties and variability related to differences in editorial policies or authorship practices across fields. By controlling factors such as editorial policies, journal scope, and authorship conventions, the study ensures that the observed gender differences reflect intrinsic patterns within dental research rather than biases related to specific specialties. Internal consistency was reinforced by the uniform application of data collection procedures, gender identification methods, and authorship contribution classifications across all analyzed journals. Future studies, including journals from other specialties, could provide broader external validation and allow for comparisons across disciplines, further contextualizing the present results.

This study addresses only one perspective of power relations (authors' contributions), and future research should incorporate an intersectional approach that considers multiple axes of identity and inequality. This broader approach would provide a more comprehensive understanding of inequities and power relations in academic authorship and research activity. The findings indicate that the contributions of the last authors were concentrated on supervisory functions, and there was no association with gender. In contrast, the first authorship displayed a greater diversity of contributions, and data collection was more frequently performed by women. 

## CONCLUSION

The findings highlight gender inequities in patterns of contributions and authorship positions, with men predominantly occupying first and last authorship roles. Men were more frequently listed as first and last authors. Gender differences in research contributions were observed only among first authors, particularly in data collection and engagement in all contribution categories. No gender differences were found in the contribution patterns of the last authors.
